# Non-Linear Response of Cable-Mass-Spring System in High-Rise Buildings under Stochastic Seismic Excitation

**DOI:** 10.3390/ma14226858

**Published:** 2021-11-14

**Authors:** Hanna Weber, Stefan Kaczmarczyk, Radosław Iwankiewicz

**Affiliations:** 1 Department of Theory of Structures, Faculty of Civil and Environmental Engineering, West Pomeranian University of Technology in Szczecin, Al. Piastów 17, 70-310 Szczecin, Poland; Radoslaw.Iwankiewicz@zut.edu.pl; 2 Faculty of Arts, Science and Technology, University of Northampton, University Dr, Northampton NN1 5PH, UK; Stefan.Kaczmarczyk@northampton.ac.uk

**Keywords:** stochastic dynamics, seismic vibrations, non-linear system, equivalent linearization technique, Gaussian white noise process

## Abstract

In high-rise buildings earthquake ground motions induce bending deformation of the host structure. Large dynamic displacements at the top of the building can be observed which in turn lead to the excitation of the cables/ropes within lift installations. In this paper, the stochastic dynamics of a cable with a spring-damper and a mass system deployed in a tall cantilever structure under earthquake excitation is considered. The non-linear system is developed to describe lateral displacements of a vertical cable with a concentrated mass attached at its lower end. The system is moving slowly in the vertical direction. The horizontal displacements of the main mass are constrained by a spring-viscous damping element. The earthquake ground motions are modelled as a filtered Gaussian white noise stochastic process. The equivalent linearization technique is then used to replace the original non-linear system with a linear one with the coefficients determined by utilising the minimization of the mean-square error between both systems. Mean values, variances and covariances of particular random state variables have been obtained by using the numerical calculation. The received results were compared with the deterministic response of the system to the harmonic process and were verified against results obtained by Monte Carlo simulation.

## 1. Introduction

High-rise buildings are very sensitive to dynamic loads because of their slenderness. The time-dependent types of loading that have a significant influence on this type of the structures are wind and earthquakes. Due to their nature they should be treated as non-deterministic forces. Dynamic wind loads or earthquakes lead to large sway motions of high-rise buildings. If the displacements at the top of the structure are significant, it may lead to damage of structural elements and, in extreme cases, to the building being destroyed. Therefore, in the design process we need to satisfy all requirements to make sure that the structure is safe. Designers are constantly trying to increase the load-bearing capacity of such systems and the comfort of people staying inside through the use of innovative solutions and materials or methods of monitoring. Some examples can be found in [1,2].

In high-rise buildings, an efficient system for transporting people and equipment should also be provided due to the significant height of the object. The sway of the structure caused by dynamic loads leads to the excitation of the cables inside the lift installation. As a result, the transverse and longitudinal vibration of the cable together with the horizontal and vertical displacements of the lift car can be observed. Lift cables are made of high-strength steel wires that are twisted together to form a strand structure. In the resonance region the significant vibrations may disrupt operation of the lift system, result in fatigue of the material or even damage of the rope. In high-rise buildings, special structural elements, such as spring-viscous damping elements or tuned-mass dampers, are used to limit the negative effects in the lift’s systems caused by vibrations. However, their correct design depends on a careful examination of the structural system behavior and an estimation of the risk of resonance. If we wish to verify the fatigue resistance of cables or their load-bearing capacity we need to be able to define the response of the lift installation to the dynamic excitation. Very often, external influences are changeable and in the design process the worst-case scenario has to be found. This is why the methods of computation that can quickly give results for structures under stochastic loads are so important.

Various models describing the behavior of the lift installation under deterministic and stochastic dynamic loads can be found for example in [3,4]. Earthquakes can bring similar problems in high-rise building that can cause the damage inside the lift installation [5,6,7]. Even if the source of the long-period ground motion is in great distance from the building it can lead to significant displacements of its base in the resonance region [8]. In [9,10] the problem of the cable-mass system dynamic response to the earthquake excitation idealized as deterministic harmonic process was considered. However due to the nature of the phenomenon the ground motion should be dealt with by stochastic methods. In the presented paper, the proposed non-linear model of cable-mass-spring system is assumed in the form of a cable with a concentrated mass attached to its lower end. The system is moving slowly in the vertical direction. The horizontal displacements of the mass are constrained by the viscous-damping element. The transverse vibrations of the cable are coupled with the longitudinal displacements. The earthquake excitation of the building base is modelled as a filtered Gaussian white noise stochastic process.

Analytical solution of non-stationary and non-linear problems is usually difficult [11]. Therefore, approximate methods and numerical techniques are often applied. One of the techniques that is commonly used to determine the stochastic response of nonlinear systems in different problems is the Monte Carlo direct simulation technique [12,13,14,15]. However, to obtain reliable results, this technique requires usually the generation of a large ensemble of excitation and response sample functions, hence the computational cost may be very high. Therefore, in this paper, the approximate analytical technique is developed, where the nonlinear model is replaced by the approximate linear one by using the equivalent (statistical) linearization technique. The particular coefficients of an equivalent linear system expressed in terms of expectations of non-linear functions of random state variables are obtained from the condition of mean-square minimization of the error between both systems. An implementation of equivalent linearization technique to obtain the response of a simplified model of lift installation under stochastic wind load can be found in [16].

The equivalent statistical linearization technique was first used to consider the non-linear random vibrations by Caughey [17]. Since that time, the technique has been implemented in many problems of stochastic dynamics, for example, [18,19,20,21]. The statistical linearization technique has been applied to solving various problems in systems under different random excitations, not only Gaussian, for example, [22]. It has also been combined with other advanced methods and techniques to conduct analysis in various domains of stochastic dynamics and more. Some examples can be found, for example, in [23,24,25,26,27,28].

The equivalent linearization technique is relatively easy to apply and allows us to obtain sufficiently accurate estimates of the mean values, variances and covariances of particular random response state variables. The advantages of this approach and its effectiveness are discussed in the present paper on the basis of a comparison with the results of the solution of an original set of nonlinear differential equations governing the behavior of the system under an idealized deterministic harmonic excitation. Also, the obtained approximate results are verified against Monte Carlo simulation for the filtered Gaussian white noise process excitation.

## 2. Materials and Methods

### 2.1. Schematic Cable-Mass-Spring Model

**Figure 1:**
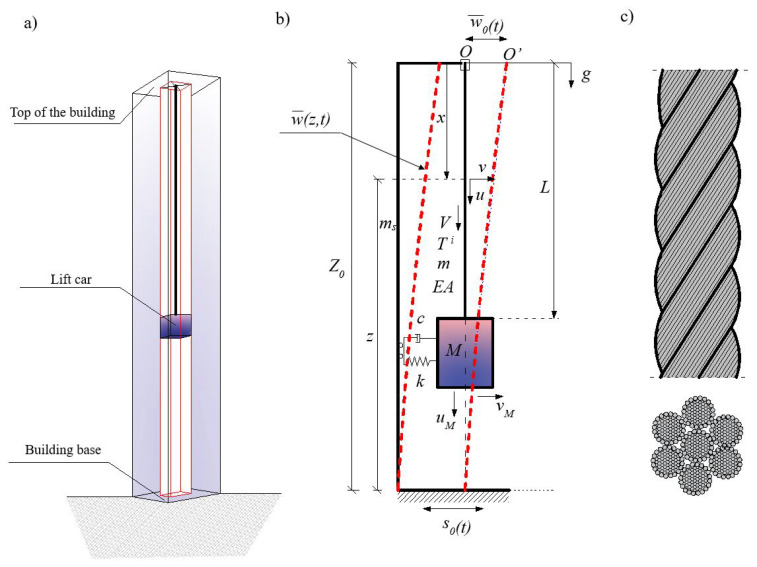
(**a**) High-rise building with lift installation; (**b**) Schematic model of cable-mass-spring system [9]; (**c**) Lift cable structure.

Consider a simplified model of a lift system represented by a cable-mass-spring system presented in Figure 1b is considered. The cantilever host structure of the total height $Z_{0}$ is exposed to the ground motion $s_{0}\left(t\right)$. The system is moving downward with the transport speed and acceleration defined as *V* and *a*, respectively. The length of the cable *L* is changing during the motion. The main mass *M* attached to the bottom end of the cable is constrained in the horizontal direction by a spring-viscous damping element with the coefficients of stiffness and damping denoted as *k* and *c*, respectively. The lateral displacements of the main mass *M* are defined as $v_{M}\left(t\right)$ while the longitudinal as $u_{M}\left(t\right)$. The ground motion leads to a sway of the structure, as a result the elastic bending deformations $\bar{w}\left(z,t\right)$ can be observed. The displacements at the top of the structure, where the $z=Z_{0}$, are denoted as ${\bar{w}}_{0}\left(t\right)=\bar{w}\left(Z_{0},t\right)$ and lead to the excitation of the rope inside the lift installation. The lateral vibrations of the cable v(x,t) are coupled with the longitudinal dynamic displacements u(x,t), where *x* is defined as the Eulerian spatial coordinate. The cable inside the lift installation is made from the high-strength steel wires that are twisted together automatically (see Figure 1c). Its mass per unit length, cross-sectional area and modulus of elasticity are assumed as *m*, *A* and *E*, respectively. The mean quasi-static tension inside the cable is defined by $T^{i}=\left[M+m\left(L-x\right)\right]\left(g-a\right)$.

### 2.2. Non-Linear System

First, let us consider the dynamic response of the system to a base motion $s_{0}\left(t\right)$. The overall (absolute) displacements w(z,t) of the structure are governed by the equation of motion:

(1) $$m_{s}\left(z\right)w_{tt}+Cw_{t}+Lw=0,$$

where $m_{s}\left(z\right)$, C, L are the linear mass density of the structure, damping operator and spatial operator related to the elastic potential energy, respectively. The symbol ${\left(\right)}_{t}$ denotes the partial derivative with respect to time.

The overall displacements of the structure presented in Figure 1b can be given by the formula:

(2) $$w\left(z,t\right)=\bar{w}\left(z,t\right)+s_{0}\left(t\right),$$

The first and second partial derivatives with respect to time are obtained as:

(3) $$\begin{array}{ccc} w_{t} & = & {\bar{w}}_{t}+{\dot{s}}_{0}\left(t\right)\\ w_{tt} & = & {\bar{w}}_{tt}+{\ddot{s}}_{0}\left(t\right). \end{array}$$

Using Equations (2) and (3) in Equation (1) brings:

(4) $$m_{s}\left(z\right){\bar{w}}_{tt}+C{\bar{w}}_{t}+L\bar{w}=-m_{s}\left(z\right){\ddot{s}}_{0}\left(t\right)-C{\dot{s}}_{0}\left(t\right)-Ls_{0}\left(t\right).$$

Since $s_{0}\left(t\right)$ is not a function of geometric co-ordinates, $Ls_{0}\left(t\right)=0$. If damping only depends on the relative motion and it does not depend on the absolute motion, then $C{\dot{s}}_{0}\left(t\right)=0$. Therefore, Equation (4) can be rewritten to the following form:

(5) $$m_{s}\left(z\right){\bar{w}}_{tt}+C{\bar{w}}_{t}+L\bar{w}=-m_{s}\left(z\right){\ddot{s}}_{0}\left(t\right),$$

whose solution can be assumed in the form of modal expansion:

(6) $$\bar{w}=\sum_{n=1}^{\infty }W_{n}\left(z\right)p_{n}\left(t\right).$$

$W_{n}\left(z\right)$ and $p_{n}\left(t\right)$ are the eigenfunctions of the structure and natural (modal) coordinates, respectively. The first and second partial derivatives of Equation (6) with respect to time are given by:

(7) $$\begin{array}{ccc} {\bar{w}}_{t} & = & \sum_{n=1}^{\infty }W_{n}\left(z\right){\dot{p}}_{n}\left(t\right),\\ {\bar{w}}_{tt} & = & \sum_{n=1}^{\infty }W_{n}\left(z\right){\ddot{p}}_{n}\left(t\right). \end{array}$$

Using Equations (6) and (7) in Equation (5) results in:

(8) $$m_{s}\left(z\right)\sum_{n=1}^{\infty }W_{n}\left(z\right){\ddot{p}}_{n}\left(t\right)+C\sum_{n=1}^{\infty }W_{n}\left(z\right){\dot{p}}_{n}\left(t\right)+L\sum_{n=1}^{\infty }W_{n}\left(z\right)p_{n}\left(t\right)=-m_{s}\left(z\right){\ddot{s}}_{0}\left(t\right).$$

Multiplying Equation (8) by the $W_{r}$ brings:

(9) $$m_{s}\left(z\right)\sum_{n=1}^{\infty }W_{n}\left(z\right)W_{r}\left(z\right){\ddot{p}}_{n}\left(t\right)+C\sum_{n=1}^{\infty }W_{n}\left(z\right)W_{r}\left(z\right){\dot{p}}_{n}\left(t\right)+L\sum_{n=1}^{\infty }W_{n}\left(z\right)W_{r}\left(z\right)p_{n}\left(t\right)=-m_{s}\left(z\right){\ddot{s}}_{0}\left(t\right)W_{r}\left(z\right).$$

By integrating the result over the domain $0\leq z\leq Z_{0}$ and using the eigenfunction orthogonality conditions:

(10) $$\int_{0}^{Z_{0}}W_{n}W_{r}dx=\{\begin{array}{cc} \neq 0 & n=r\\ 0 & n\neq r \end{array}\;\;\;\;\;,$$

Equation (9) is transformed to the following form:

(11) $${\ddot{p}}_{n}\left(t\right)\int_{0}^{Z_{0}}m_{s}\left(z\right)W_{r}^{2}\left(z\right)dz+C{\dot{p}}_{n}\left(t\right)\int_{0}^{Z_{0}}W_{r}^{2}\left(z\right)dz+Lp_{n}\left(t\right)\int_{0}^{Z_{0}}W_{r}^{2}\left(z\right)dz=-{\ddot{s}}_{0}\left(t\right)\int_{0}^{Z_{0}}m_{s}\left(z\right)W_{r}\left(z\right)dz.$$

Dividing all terms of Equation (11) by the modal mass defined by the expression:

(12) $$m_{r}=\int_{0}^{Z_{0}}m_{s}\left(z\right)W_{r}^{2}\left(z\right)dz,$$

the modal equations are obtained as:

(13) $${\ddot{p}}_{r}\left(t\right)+2\zeta_{r}\omega_{r}{\dot{p}}_{r}\left(t\right)+\omega_{r}^{2}p_{r}\left(t\right)=-\frac{{\ddot{s}}_{0}\left(t\right)}{m_{r}}\int_{0}^{Z_{0}}m_{s}\left(z\right)W_{r}\left(z\right)dz=P_{r}\left(t\right),$$

where $\omega_{r}$ and $\zeta_{r}$ denote the natural frequencies and modal damping ratios, respectively [9]. $P_{r}\left(t\right)$ are the modal excitation terms. If the natural frequencies of damped vibrations are determined as $\omega_{dr}=\omega_{r}\sqrt{1-\zeta_{r}^{2}}$, the steady-state response of the structure is defined by:

(14) $$P_{r}\left(t\right)=\frac{1}{\omega_{dr}}\int_{0}^{t}P_{r}\left(t-\tau \right)e^{-\zeta_{r}\omega_{r}\tau }sin\left(\omega_{dr}\tau \right)d\tau .$$

If we are looking for an approximate solution of the Equation (5), we can assume that: [29]

(15) $$\bar{w}\left(z,t\right)=\sum_{n=1}^{N}\Psi_{n}\left(z\right)p_{n}\left(t\right),$$

where $\Psi_{n}$ are the approximating functions and $p_{n}\left(t\right)$ denote the general coordinates that correspond to the lateral motions of the structure. The first and second partial derivatives of Equation (15) with respect to time are given by:

(16) $$\begin{array}{ccc} {\bar{w}}_{t} & = & \sum_{n=1}^{N}\Psi_{n}\left(z\right){\dot{p}}_{n}\left(t\right),\\ {\bar{w}}_{tt} & = & \sum_{n=1}^{N}\Psi_{n}\left(z\right){\ddot{p}}_{n}\left(t\right). \end{array}$$

Using Equation (16) in Equation (5) leads to:

(17) $$m_{s}\left(z\right)\sum_{n=1}^{N}\Psi_{n}\left(z\right){\ddot{p}}_{n}\left(t\right)+C\sum_{n=1}^{N}\Psi_{n}\left(z\right){\dot{p}}_{n}\left(t\right)+L\sum_{n=1}^{N}\Psi_{n}\left(z\right)p_{n}\left(t\right)=-m_{s}\left(z\right){\ddot{s}}_{0}\left(t\right).$$

Multiplying both sides of Equation (17) by $\Psi_{r}$ and integrating the result over the domain $0\leq z\leq Z_{0}$ brings:

(18) $$\begin{array}{lll} & & \int_{0}^{Z_{0}}\Psi_{r}\left(z\right)m_{s}\left(z\right)\Psi_{n}\left(z\right)dz{\ddot{p}}_{n}\left(t\right)+\int_{0}^{Z_{0}}\Psi_{r}\left(z\right)C\Psi_{n}\left(z\right)dz{\dot{p}}_{n}\left(t\right)+\int_{0}^{Z_{0}}\Psi_{r}\left(z\right)L\Psi_{n}\left(z\right)dzp_{n}\left(t\right)\\ & = & -{\ddot{s}}_{0}\left(t\right)\int_{0}^{Z_{0}}m_{s}\left(z\right)\Psi_{r}\left(z\right)dz. \end{array}$$

Assuming M, C, K and F(t) as the mass, damping and stiffness matrices and the vector of generalized excitation forces, respectively, expressed by the following equations:

(19) $$\begin{array}{ccc} M & = & \left[m_{rn}\right],\;\;m_{rn}=\int_{0}^{Z_{0}}\Psi_{r}\left(z\right)m_{s}\left(z\right)\Psi_{n}\left(z\right)dz,\\ C & = & \left[c_{rn}\right],\;\;c_{rn}=\int_{0}^{Z_{0}}\Psi_{r}\left(z\right)C\Psi_{n}\left(z\right)dz,\\ K & = & \left[k_{rn}\right],\;\;k_{rn}=\int_{0}^{Z_{0}}\Psi_{r}\left(z\right)L\Psi_{n}\left(z\right)dz,\\ F(t) & = & \left[F_{r}\left(t\right)\right],\;\;F_{r}\left(t\right)=-{\ddot{s}}_{0}\left(t\right)\int_{0}^{Z_{0}}m_{s}\left(z\right)\Psi_{r}\left(z\right)d, \end{array}$$

Equation (18) is rewritten to equation of motion in the matrix form:

(20) $$M\ddot{p}\left(t\right)+C\dot{p}\left(t\right)+Kp\left(t\right)=F\left(t\right).$$

As is well known, the sway of the structure leads to the excitation of the rope inside the lift installation. The cable axial strain is assumed to be $\varepsilon =u_{x}+\frac{1}{2}v_{x}^{2}$ and the quasi-static tension of the cable is given by the expression T=(M+mL)(g−a). From the Hamilton’s Principle for external work of non-conservative forces, kinetic and potential energies of the system, the following partial differential equations of motion are obtained:

(21) $$\begin{array}{ccc} m\frac{D^{2}u}{Dt^{2}} & - & EA\varepsilon_{x}=0,\\ m\frac{D^{2}v}{Dt^{2}} & - & Tv_{xx}+m\left(g-a\right)\left(xv_{xx}+v_{x}\right)-EA{\left(\varepsilon v_{x}\right)}_{x}=0,\\ M{\ddot{v}}_{M} & + & T^{i}\left(L\right)v_{x}{|}_{x=L}+c\dot{▵}+k▵+EA\varepsilon {{|}_{x=L}v_{x}|}_{x=L}=0,\\ M{\ddot{u}}_{M} & + & {EA\varepsilon |}_{x=L}=0, \end{array}$$

where Δ denotes the deformation of the spring expressed by $\Delta =v_{M}-w_{M}$, with $w_{M}=w\left(Z_{0}-L,t\right)$ being the bending deformation of the host structure at the level of the main mass. The derivation of Equation (21) in detail can be found in [9]. The total derivatives with respect to time are given by the equations:

(22) $$\begin{array}{ccc} \frac{D(\;\;)}{Dt} & = & {\left(\;\;\right)}_{t}+V{\left(\;\;\right)}_{x},\\ \frac{D^{2}\left(\;\;\right)}{Dt^{2}} & = & {\left(\;\;\right)}_{tt}+2V{\left(\;\;\right)}_{xt}+V^{2}{\left(\;\;\right)}_{xx}+a{\left(\;\;\right)}_{x}. \end{array}$$

Because the lateral frequencies of tensioned metallic cables are much lower than the fundamental longitudinal frequencies and in the case of long-period ground motions, the excitation frequencies are much lower than the fundamental longitudinal frequencies, and the longitudinal inertia of the cable in the first equation of (21) can be neglected [30]. Integrating this equation using boundary conditions u(0,t)=0 and $u\left(L,t\right)=u_{M}\left(t\right)$ leads to the following expression [9]:

(23) $$u_{x}\left(x,t\right)=e\left(t;\tau \right)-\frac{v_{x}^{2}\left(x,t\right)}{2},$$

where e(t;τ) represents the averaged over the length quasi-static axial strain in the rope expressed by:

(24) $$e\left(t;\tau \right)=\frac{u_{M}\left(t\right)}{L(\tau )}+\frac{1}{2L(\tau )}\int_{0}^{L}v_{x}^{2}\left(x,t\right)dx.$$

This results in reducing the dynamic model described by Equation (21) to three equations of motion. The change of L(t) over a period $T_{0}$ corresponding to the fundamental frequency of the system $f_{0}$ is small in comparison to the total length of the cable [31,32], therefore the slow time scale is defined as τ=ϵt. The small parameter ϵ<<1 is expressed by the equation $\epsilon =\dot{L}\left(t_{0}\right)/f_{0}L_{0}$ [33], where $t_{0}$ denotes a given time instant corresponding to $f_{0}$ and $L_{0}=L\left(t_{0}\right)$.

### 2.3. Base Excitation and the Nonlinear Response of the Cable-Mass-Spring System

The earthquake leads to the base motion excitation. As a result, the sway of the structure can be observed that causes the bending deformations of the host structure, which is treated as a cantilever beam. In the resonance region, they can be described approximately by the polynomial shape function $\Psi \left(\eta \right)=3\eta^{2}-2\eta^{3}$, where $\eta =\frac{z}{Z_{0}}$. In this consideration, it is assumed that the impact of the cable system vibrations on the structural response can be neglected.

The time dependent overall lateral displacements of the cable-mass system can be expressed by the equation:

(25) $$v\left(x,t\right)=\bar{v}\left(x,t\right)+s_{0}\left(t\right)+(1+\frac{\Psi_{L}-1}{L(\tau )}x){\bar{w}}_{0}\left(t\right),$$

where $\Psi_{L}$ and ${\bar{w}}_{0}\left(t\right)$ are adopted in the form:

(26) $$\begin{array}{ccc} \Psi_{L}=\Psi (\frac{Z_{0}-L\left(\tau \right)}{Z_{0}}) & \mathrm{and} & {\bar{w}}_{0}\left(t\right)=\bar{w}\left(Z_{0},t\right). \end{array}$$

The relative lateral displacements can be described by the finite series as:

(27) $$\bar{v}\left(x,t;\tau \right)=\sum_{n=1}^{N}\Phi_{n}\left[x;L\left(\tau \right)\right]q_{n}\left(t\right),$$

with orthogonal trial functions expressed by the formula $\Phi_{n}\left[x,L\left(\tau \right)\right]=\sin\left[\sigma_{n}\left(L\left(\tau \right)\right)x\right],$n=1,2,⋯,N where *N* denotes the number of considered modes. The $q_{n}$ describes the generalized coordinates that correspond to the lateral motions of the cable system. The eigenvalues $\sigma_{n}\left(\tau \right)$ are slow varying and can be defined by the equations:

(28) $$(k-\frac{M}{m}T_{M}\sigma_{n}^{2})sin\left(\sigma_{n}L\right)+T_{M}\sigma_{n}cos\left(\sigma_{n}L\right)=0,\;\;\;T_{M}\equiv T^{i}\left(L\right)=\left(M+mL\right)\left(g-a\right).$$

Using Equations (25) and (27) in the reduced system of Equation (21) leads to the differential equations in the form [9]:

(29) $$\begin{array}{c} {\ddot{q}}_{r}+2{\tilde{\zeta }}_{r}{\tilde{\omega }}_{r}{\dot{q}}_{r}+\sum_{n=1}^{N}C_{rn}{\dot{q}}_{n}+{\tilde{\omega }}_{r}^{2}q_{r}+\sum_{i=1}^{N}[K_{rn}+\frac{EA}{{\tilde{m}}_{r}}(\frac{\Psi_{L}-1}{L}{\bar{w}}_{0}{)}^{2}(\frac{1}{L}A_{rn}-\frac{1}{2}\Gamma_{rn})]q_{n}+\frac{EA}{{\tilde{m}}_{r}L}\frac{\Psi_{L}-1}{L}\alpha_{r}{\bar{w}}_{0}u_{M}\\ =\frac{EA}{{\tilde{m}}_{r}L}[\sum_{n=1}^{N}\Gamma_{rn}q_{n}u_{M}+\frac{1}{2}\frac{\Psi_{L}-1}{L}{\bar{w}}_{0}\sum_{i=1}^{N}\sum_{j=1}^{N}\left(2\alpha_{i}\Gamma_{rj}-\alpha_{r}\kappa_{ij}\right)q_{i}q_{j}+\frac{1}{2}\sum_{i=1}^{N}\sum_{j=1}^{N}\sum_{k=1}^{N}\Gamma_{rk}\kappa_{ij}q_{i}q_{j}q_{k}]+{\tilde{Q}}_{r}\left(t;\tau \right),\\ {\ddot{u}}_{M}+2\zeta_{M}\omega_{M}{\dot{u}}_{M}+\omega_{M}^{2}u_{M}+\frac{EA}{ML}\frac{\Psi_{L}-1}{L}{\bar{w}}_{0}\sum_{n=1}^{N}\alpha_{n}q_{n}=-\frac{1}{2}\frac{EA}{ML}\sum_{i=1}^{N}\sum_{j=1}^{N}\kappa_{ij}q_{i}q_{j}-\frac{EA}{2M}(\frac{\Psi_{L}-1}{L}{\bar{w}}_{0}{)}^{2}, \end{array}$$

where the symbols ${\tilde{\zeta }}_{r}$ and $\zeta_{M}$ denote the damping ratios for the cable lateral mode and for the mass longitudinal mode, respectively. The particular expressions included in Equation (29) are defined by:

(30) $$\begin{array}{ccc} {\tilde{\omega }}_{r}^{2} & = & \sigma_{r}^{2}\frac{T}{m};\;\;\alpha_{r}=\Phi_{r}\left(L\right);\;\;{\tilde{m}}_{r}=m\int_{0}^{L}\Phi_{r}^{2}dx+M\alpha_{r}^{2};\;\;A_{rn}=\alpha_{r}\alpha_{n};\\ K_{rn} & = & \frac{m}{{\tilde{m}}_{r}}[g\Psi_{rn}+\left[V^{2}-L\left(g-a\right)\right]\gamma_{rn}+\left(g-a\right)\Theta_{rn}];\;\;C_{rn}=2\frac{m}{{\tilde{m}}_{r}}V\Psi_{rn}+\frac{1}{{\tilde{m}}_{r}}cA_{rn};\\ \Theta_{rn} & = & \int_{0}^{L}x\Phi_{n}^{"}\Phi_{r}dx;\;\;\Psi_{rn}=\int_{0}^{L}\Phi_{n}^{\prime }\Phi_{r}dx;\;\;\Upsilon_{rn}=\int_{0}^{L}\Phi_{n}^{"}\Phi_{r}dx;\;\;\kappa_{ij}=\int_{0}^{L}\Phi_{i}^{\prime }\Phi_{j}^{\prime }dx;\;\;\Gamma_{rn}=\Upsilon_{rn}-\alpha_{r}\Phi_{n}^{\prime }\left(L\right);\\ {\tilde{Q}}_{r}\left(t;\tau \right) & = & \frac{1}{{\tilde{m}}_{r}}\{-\left(m\chi_{r}+M\alpha_{r}\right){\ddot{s}}_{0}-[m(\chi_{r}+\frac{\Psi_{L}-1}{L}\Pi_{r})+M\Psi_{L}\alpha_{r}]{\ddot{\bar{w}}}_{0}-2mV\frac{\Psi_{L}-1}{L(\tau )}\chi_{r}{\dot{\bar{w}}}_{0}+\\ & & -\frac{\Psi_{L}-1}{L(\tau )}\left(mg\chi_{r}+T_{M}\alpha_{r}\right){\bar{w}}_{0}-\frac{EA}{2}\alpha_{r}(\frac{\Psi_{L}-1}{L(\tau )}{\bar{w}}_{0}{)}^{3}\};\\ \chi_{r} & = & \int_{0}^{L}\Phi_{r}dx;\;\;\Pi_{r}=\int_{0}^{L}x\Phi_{r}dx. \end{array}$$

To describe the system’s behavior in the resonance region, a single-mode approximation for r-th mode with displacements assumed in the form presented by Equation (27) can be considered. Then, the equations of motion are obtained as:

(31) $$\begin{array}{c} {\ddot{q}}_{r}+{\tilde{c}}_{r}{\dot{q}}_{r}+{\tilde{\omega }}_{r}^{2}q_{r}+{\tilde{k}}_{r}q_{r}-\frac{EA}{{\tilde{m}}_{r}}\bar{e}[\Gamma_{rr}q_{n}-\frac{\Psi_{L}-1}{L}\alpha_{r}{\bar{w}}_{0}]=-\frac{1}{{\tilde{m}}_{r}}\{\left(m\chi_{r}+M\alpha_{r}\right){\ddot{s}}_{0}\\ +[m(\chi_{r}+\frac{\Psi_{L}-1}{L}\Pi_{r})+M\Psi_{L}\alpha_{r}]{\ddot{\bar{w}}}_{0}+2mV\frac{\Psi_{L}-1}{L(\tau )}\chi_{r}{\dot{\bar{w}}}_{0}+\frac{\Psi_{L}-1}{L(\tau )}\left(mg\chi_{r}+T_{M}\alpha_{r}\right){\bar{w}}_{0}\},\\ {\ddot{u}}_{M}+2\zeta_{M}\omega_{M}{\dot{u}}_{M}+\frac{EA}{M}\bar{e}=0, \end{array}$$

where

(32) $$\begin{array}{ccc} {\tilde{c}}_{r} & = & 2{\tilde{\zeta }}_{r}{\tilde{\omega }}_{r}+2\frac{m}{{\tilde{m}}_{r}}V\Psi_{rr}+\frac{1}{{\tilde{m}}_{r}}c\alpha_{r}^{2};\\ {\tilde{k}}_{r} & = & \frac{m}{{\tilde{m}}_{r}}[g\Psi_{rr}+\left[V^{2}-L\left(g-a\right)\right]\gamma_{rr}+\left(g-a\right)\Theta_{rr}];\\ \bar{e} & = & \frac{u_{M}\left(t\right)}{L(\tau )}+\frac{1}{2L}\kappa_{rr}\left(\tau \right)q_{r}^{2}\left(t\right)+\frac{\Psi_{L}-1}{L^{2}\left(\tau \right)}{\bar{w}}_{0}\alpha_{r}\left(\tau \right)q_{r}\left(t\right)+\frac{1}{2}(\frac{\Psi_{L}-1}{L(\tau )}{)}^{2}{\bar{w}}_{0}^{2}\left(t\right);\\ \omega_{M} & = & \sqrt{\frac{EA}{ML}}. \end{array}$$

### 2.4. Stochastic Modelling of the Ground Motion Due to an Earthquake

**Figure 2:**
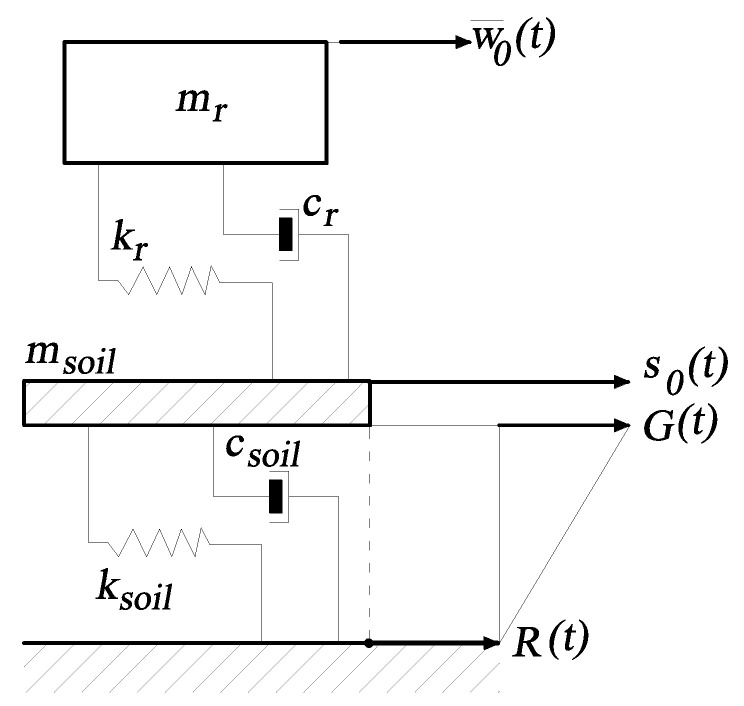
An idealized model of building with stochastic ground motion.

The structure, due to an earthquake, is subjected to a base motion $s_{o}\left(t\right)$, which is represented as (see Figure 2):

(33) $$s_{o}\left(t\right)=G\left(t\right)+R\left(t\right),$$

where G(t) is the ground motion relative to the bedrock and R(t) is the absolute motion of the bedrock. Between the bedrock and the ground surface there is a layer of soil. The properties of the soil layer are idealized as a single-degree-of-freedom system with the mass $m_{soil}$, stiffness coefficient $k_{soil}=\omega_{s}^{2}m_{soil}$ and the damping coefficient $c_{soil}=2\zeta_{s}\omega_{s}m_{soil}$.

In the model presented in Figure 2 subscript *r* and corresponding modal mass $m_{r}$, given as:

(34) $$m_{r}=\int_{0}^{Z_{0}}m_{s}\left(z\right)W_{r}^{2}\left(z\right)dz,$$

refer to the fundamental mode of the structure, where $m_{s}$ denotes the linear mass density of the structure. Using the approximations given by the equation:

(35) $$m_{s}\left(z\right)\approx {\tilde{m}}_{s}=const,$$

and considering the response of the structure in the fundamental resonance, the fundamental modal shape function is expressed by the polynomial shape function $W_{r}\left(z\right)\approx \Psi \left(\eta \right)$. The modal mode approach leads to the formula (see Equation (13)):

(36) $${\ddot{p}}_{r}\left(t\right)+2\zeta_{r}\omega_{r}{\dot{p}}_{r}\left(t\right)+\omega_{r}^{2}p_{r}\left(t\right)=-{\ddot{s}}_{0}\left(t\right)\frac{{\tilde{m}}_{s}}{m_{r}}\int_{0}^{Z_{0}}\Psi \left(\eta \right)dz,$$

therefore, the displacements at the top end of the structure are defined as $\bar{w}\left(Z_{0},t\right)={\bar{w}}_{0}\left(t\right)=p_{r}\left(t\right)$ and consequently ${\dot{\bar{w}}}_{0}\left(t\right)={\dot{p}}_{r}\left(t\right)$, ${\ddot{\bar{w}}}_{0}\left(t\right)={\ddot{p}}_{r}\left(t\right)$. The model shown in Figure 2 represents a 2DOF system, where the ground motion is coupled with motion of the building structure and is as follows:

(37) $$m_{soil}{\ddot{s}}_{0}+c_{soil}\left({\dot{s}}_{0}-\dot{R}\right)-c_{r}\left({\dot{w}}_{0}-{\dot{s}}_{0}\right)+k_{soil}\left(s_{0}-R\right)-k_{r}\left(w_{0}-s_{0}\right)=0.$$

Noting that $w_{0}={\bar{w}}_{0}\left(t\right)+s_{0}=p_{r}+s_{0}$ and $s_{0}=R+G$ we obtain:

(38) $$m_{soil}\left(\ddot{R}+\ddot{G}\right)+c_{soil}\dot{G}-c_{r}{\dot{p}}_{r}+k_{soil}G-k_{r}p_{r}=0.$$

Dividing both sides of the Equation (37) by $m_{soil}$ leads to the following formula:

(39) $$\ddot{G}+\frac{c_{soil}}{m_{soil}}\dot{G}-\frac{c_{r}}{m_{soil}}{\dot{p}}_{r}+\frac{k_{soil}}{m_{soil}}G-\frac{k_{r}}{m_{soil}}p_{r}=-\ddot{R}.$$

If we assume that the ground motion is not influenced by the building structure we get:

(40) $$\ddot{G}+\frac{c_{soil}}{m_{soil}}\dot{G}+\frac{k_{soil}}{m_{soil}}G=-\ddot{R}.$$

Noting that $\frac{c_{soil}}{m_{soil}}=2\zeta_{s}\omega_{s}$ and $\frac{k_{soil}}{m_{soil}}=\omega_{s}^{2}$ the Equation (40) is transformed to the following form:

(41) $$\ddot{G}\left(t\right)+2\zeta_{s}\omega_{s}\dot{G}\left(t\right)+\omega_{s}^{2}G\left(t\right)=-\ddot{R}\left(t\right).$$

The negative acceleration $-\ddot{R}\left(t\right)$ of the bedrock is assumed as a Gaussian white noise ξ(t), hence:

(42) $$\ddot{G}\left(t\right)+2\zeta_{s}\omega_{s}\dot{G}\left(t\right)+\omega_{s}^{2}G\left(t\right)=\sqrt{2\pi P}\xi \left(t\right).$$

Stochastic differential equations governing the state vector $G={\left[G_{1},G_{2}\right]}^{T}$ are:

(43) $$\begin{array}{ccc} dG_{1}\left(t\right) & = & G_{2}\left(t\right)dt,\\ dG_{2}\left(t\right) & = & -2\zeta_{s}\omega_{s}G_{2}dt-\omega_{s}^{2}G_{1}dt+\sqrt{2\pi P}dW\left(t\right), \end{array}$$

where W(t) is a Wiener process and *P* is the constant spectral density of the white noise process ξ(t). The state vector G must be appended to the state vector of the dynamic system and in the equations of motion the following replacement must be done:

(44) $$-{\ddot{s}}_{0}\left(t\right)=\omega_{s}^{2}G_{1}+2\zeta_{s}\omega_{s}G_{2}.$$

Using Equation (36) together with the Equation (44) in Equation (31) leads to a set of equations of motion given by:

(45) $$\begin{array}{c} {\ddot{q}}_{r}+{\tilde{c}}_{r}{\dot{q}}_{r}+{\tilde{\omega }}_{r}^{2}q_{r}+{\tilde{k}}_{r}q_{r}-\frac{EA}{{\tilde{m}}_{r}}\bar{e}[\Gamma_{rr}q_{n}-\frac{\Psi_{L}-1}{L}\alpha_{r}p_{r}]=-\frac{1}{{\tilde{m}}_{r}}\{-\left(m\chi_{r}+M\alpha_{r}\right)\left(\omega_{s}^{2}G_{1}+2\zeta_{s}\omega_{s}G_{2}\right)\\ +[m(\chi_{r}+\frac{\Psi_{L}-1}{L}\Pi_{r})+M\Psi_{L}\alpha_{r}](-2\zeta_{r}\omega_{r}{\dot{p}}_{r}\left(t\right)-\omega_{r}^{2}p_{r}\left(t\right)+\left(\omega_{s}^{2}G_{1}+2\zeta_{s}\omega_{s}G_{2}\right)\frac{{\tilde{m}}_{s}}{m_{r}}\int_{0}^{Z_{0}}\Psi \left(\eta \right)dz)\\ +2mV\frac{\Psi_{L}-1}{L(\tau )}\chi_{r}{\dot{p}}_{r}+\frac{\Psi_{L}-1}{L(\tau )}\left(mg\chi_{r}+T_{M}\alpha_{r}\right)p_{r}\};\\ {\ddot{u}}_{M}+2\zeta_{M}\omega_{M}{\dot{u}}_{M}+\frac{EA}{M}\bar{e}=0. \end{array}$$

### 2.5. Equivalent Linearization Technique Implementation

Converting the second-order differential equations to the first order differential ones is performed by using the following expression:

(46) dY(t)=c(Y(t),t)dt+d(t)dW(t),

where W(t) denotes the standard Wiener process, c(Y(t),t) is the drift vector and d(t) means the diffusion vector. The augmented state vector Y(t) is defined by:

(47) $$Y\left(t\right)={\left[\begin{array}{cccccccc} q_{r}\left(t\right) & {\dot{q}}_{r}\left(t\right) & p_{r}\left(t\right) & {\dot{p}}_{r}\left(t\right) & u_{M}\left(t\right) & {\dot{u}}_{M}\left(t\right) & G_{1}\left(t\right) & G_{2}\left(t\right) \end{array}\right]}^{T}.$$

The particular elements of drift vector are expressed as:

(48) $$\begin{array}{ccc} c_{1}\left(Y\left(t\right)\right) & = & {\dot{q}}_{r}\left(t\right);\\ c_{2}\left(Y\left(t\right)\right) & = & -{\tilde{c}}_{r}{\dot{q}}_{r}\left(t\right)-{\tilde{\omega }}_{r}^{2}q_{r}\left(t\right)-{\tilde{k}}_{r}q_{r}\left(t\right)+\frac{EA}{{\tilde{m}}_{r}}\bar{e}\left(\tau ;t\right)[\Gamma_{rr}q_{n}\left(t\right)-\frac{\Psi_{L}-1}{L(\tau )}\alpha_{r}\left(\tau \right)p_{r}\left(t\right)]\\ & & -\frac{1}{{\tilde{m}}_{r}}\{-\left(m\chi_{r}+M\alpha_{r}\left(\tau \right)\right)\left(\omega_{s}^{2}G_{1}\left(t\right)+2\zeta_{s}\omega_{s}G_{2}\left(t\right)\right)+[m(\chi_{r}+\frac{\Psi_{L}-1}{L(\tau )}\Pi_{r})+M\Psi_{L}\alpha_{r}\left(\tau \right)]\\ & & \times (-2\zeta_{r}\omega_{r}{\dot{p}}_{r}\left(t\right)-\omega_{r}^{2}p_{r}\left(t\right)+\left(\omega_{s}^{2}G_{1}\left(t\right)+2\zeta_{s}\omega_{s}G_{2}\left(t\right)\right)\frac{{\tilde{m}}_{s}}{m_{r}}\int_{0}^{Z_{0}}\Psi \left(\eta \right)dz)\\ & & +2mV\frac{\Psi_{L}-1}{L(\tau )}\chi_{r}{\dot{p}}_{r}\left(t\right)+\frac{\Psi_{L}-1}{L(\tau )}\left(mg\chi_{r}+T_{M}\alpha_{r}\left(\tau \right)\right)p_{r}\left(t\right)\};\\ c_{3}\left(Y\left(t\right)\right) & = & {\dot{p}}_{r}\left(t\right);\\ c_{4}\left(Y\left(t\right)\right) & = & -2\zeta_{r}\omega_{r}{\dot{p}}_{r}\left(t\right)-\omega_{r}^{2}p_{r}\left(t\right)+\left(\omega_{s}^{2}G_{1}\left(t\right)+2\zeta_{s}\omega_{s}G_{2}\left(t\right)\right)\frac{{\tilde{m}}_{s}}{m_{r}}\int_{0}^{Z_{0}}\Psi \left(\eta \right)dz;\\ c_{5}\left(Y\left(t\right)\right) & = & {\dot{u}}_{M}\left(t\right);\\ c_{6}\left(Y\left(t\right)\right) & = & -2\zeta_{M}\omega_{M}{\dot{u}}_{M}\left(t\right)-\frac{EA}{M}\bar{e}\left(\tau ;t\right);\\ c_{7}\left(Y\left(t\right)\right) & = & G_{2}\left(t\right);\\ c_{8}\left(Y\left(t\right)\right) & = & -2\zeta_{s}\omega_{s}G_{2}\left(t\right)-\omega_{s}^{2}G_{1}\left(t\right). \end{array}$$

The augmented state vector transformation to the centralized state vector is required to convert the original nonlinear set of differential equation into the linear one by using the equivalent linearization technique. The centralized state vector is defined as:

(49) $$Y^{0}\left(t\right)={\left[\begin{array}{cccccccc} Y_{1}^{0}\left(t\right) & Y_{2}^{0}\left(t\right) & Y_{3}^{0}\left(t\right) & Y_{4}^{0}\left(t\right) & Y_{5}^{0}\left(t\right) & Y_{6}^{0}\left(t\right) & Y_{7}^{0}\left(t\right) & Y_{8}^{0}\left(t\right) \end{array}\right]}^{T},$$

where

$$\begin{array}{cccc} Y_{1}^{0}\left(t\right)=q_{r}\left(t\right)-\mu_{q_{r}}\left(t\right); & \;\;Y_{2}^{0}\left(t\right)={\dot{q}}_{r}\left(t\right)-\mu_{{\dot{q}}_{r}}\left(t\right); & \;\;Y_{3}^{0}\left(t\right)=p_{r}\left(t\right)-\mu_{p_{r}}\left(t\right); & \;\;Y_{4}^{0}\left(t\right)={\dot{p}}_{r}\left(t\right)-\mu_{{\dot{p}}_{r}}\left(t\right);\\ Y_{5}^{0}\left(t\right)=u_{M}\left(t\right)-\mu_{u_{M}}\left(t\right); & \;\;Y_{6}^{0}\left(t\right)={\dot{u}}_{M}\left(t\right)-\mu_{{\dot{u}}_{M}}\left(t\right); & \;\;Y_{7}^{0}\left(t\right)=G_{1}\left(t\right)-\mu_{G_{1}}\left(t\right); & \;\;Y_{8}^{0}\left(t\right)=G_{2}\left(t\right)-\mu_{G_{2}}\left(t\right). \end{array}$$

The differential equations for the mean values are given by:

(50) $$\frac{d}{dt}\mu \left(t\right)=E[c\left(Y^{0}\left(t\right)\right],\;\;\mathrm{where}\;\;\mu \left(t\right)=E\left[Y\left(t\right)\right].$$

Using the centralized state vector leads to the stochastic equation in the following form:

(51) $$dY^{0}\left(t\right)=c^{0}\left(Y^{0}\left(t\right),t\right)dt+d\left(t\right)dW\left(t\right),$$

where the centralized drift vector $c^{0}\left(Y^{0}\left(t\right),t\right)$ is expressed by:

(52) $$c^{0}\left(Y^{0}\left(t\right),t\right)=c\left(Y^{0}\left(t\right),t\right)-E\left[c\left(Y^{0}\left(t\right),t\right)\right]$$

and diffusion vector, independent of the state vector, is defined as:

(53) $$d\left(t\right)={\left[\begin{array}{cccccccc} 0 & 0 & 0 & 0 & 0 & 0 & 0 & \sqrt{2\pi P} \end{array}\right]}^{T}.$$

The particular elements of vector $c^{0}\left(Y^{0}\left(t\right),t\right)$ are given by the following formulae:

(54) $$\begin{array}{ccc} c_{1}^{0}\left(Y^{0}\left(t\right)\right) & = & Y_{2}^{0}\left(t\right),\\ c_{2}^{0}\left(Y^{0}\left(t\right)\right) & = & -{\tilde{c}}_{r}Y_{2}^{0}\left(t\right)-{\tilde{\omega }}_{r}^{2}Y_{1}^{0}\left(t\right)-{\tilde{k}}_{r}Y_{1}^{0}\left(t\right)\\ & & +\frac{EA}{{\tilde{m}}_{r}}\frac{\Gamma_{rr}}{L(\tau )}(Y_{1}^{0}\left(t\right)Y_{5}^{0}\left(t\right)+Y_{1}^{0}\left(t\right)E\left[u_{M}\right]+Y_{5}^{0}\left(t\right)E\left[q_{r}\right]-E\left[Y_{1}^{0}\left(t\right)Y_{5}^{0}\left(t\right)\right])\\ & & +\frac{3EA}{2{\tilde{m}}_{r}L\left(\tau \right)}\Gamma_{rr}\kappa_{rr}\left(\tau \right)(\frac{1}{3}{\left(Y_{1}^{0}\left(t\right)\right)}^{3}+Y_{1}^{0}{\left(t\right)}^{2}E\left[q_{r}\right]+Y_{1}^{0}\left(t\right)E{\left[q_{r}\right]}^{2}-E\left[Y_{1}^{0}{\left(t\right)}^{2}\right]E\left[q_{r}\right]-E\left[Y_{1}^{0}\left(t\right)\right]E{\left[q_{r}\right]}^{2})\\ & & +\frac{EA}{{\tilde{m}}_{r}}\frac{\Psi_{L}-1}{L^{2}\left(\tau \right)}\alpha_{r}\left(\tau \right)\left(\Gamma_{rr}-\frac{1}{2}\kappa_{rr}\left(\tau \right)\right)({\left(Y_{1}^{0}\left(t\right)\right)}^{2}Y_{3}^{0}\left(t\right)+2Y_{1}^{0}\left(t\right)Y_{3}^{0}\left(t\right)E\left[q_{r}\right]+Y_{3}^{0}\left(t\right){\left(E\left[q_{r}\right]\right)}^{2}\\ & & +{\left(Y_{1}^{0}\left(t\right)\right)}^{2}E\left[p_{r}\left(t\right)\right]+2Y_{1}^{0}\left(t\right)E\left[q_{r}\right]E\left[p_{r}\left(t\right)\right]-2E\left[Y_{1}^{0}\left(t\right)Y_{3}^{0}\left(t\right)\right]E\left[q_{r}\right]-E\left[{\left(Y_{1}^{0}\left(t\right)\right)}^{2}\right]E\left[p_{r}\left(t\right)\right])\\ & & +\frac{EA}{{\tilde{m}}_{r}}(\frac{\Psi_{L}-1}{L(\tau )}{)}^{2}(\frac{1}{2}\Gamma_{rr}-\frac{\alpha_{r}^{2}\left(\tau \right)}{L})({\left(Y_{3}^{0}\left(t\right)\right)}^{2}Y_{1}^{0}\left(t\right)+{\left(Y_{3}^{0}\left(t\right)\right)}^{2}E\left[q_{r}\right]+2Y_{3}^{0}\left(t\right)Y_{1}^{0}\left(t\right)E\left[p_{r}\right]\\ & & +2Y_{3}^{0}\left(t\right)E\left[p_{r}\right]E\left[q_{r}\right]+Y_{1}^{0}\left(t\right){\left(E\left[p_{r}\right]\right)}^{2}-E\left[{\left(Y_{3}^{0}\left(t\right)\right)}^{2}\right]E\left[q_{r}\right]-2E\left[Y_{3}^{0}\left(t\right)Y_{1}^{0}\left(t\right)\right]E\left[p_{r}\right])\\ & & -\frac{EA}{{\tilde{m}}_{r}}\frac{\Psi_{L}-1}{L^{2}\left(\tau \right)}\alpha_{r}\left(\tau \right)\left(Y_{3}^{0}\left(t\right)Y_{5}^{0}\left(t\right)+Y_{3}^{0}\left(t\right)E\left[u_{M}\left(t\right)\right]+Y_{5}^{0}\left(t\right)E\left[p_{r}\right]-E\left[Y_{3}^{0}\left(t\right)Y_{5}^{0}\left(t\right)\right]\right)\\ & & -\frac{EA}{{\tilde{m}}_{r}}\frac{3}{2}(\frac{\Psi_{L}-1}{L(\tau )}{)}^{3}\alpha_{r}\left(\tau \right)\left(\frac{1}{3}{\left(Y_{3}^{0}\left(t\right)\right)}^{3}+{\left(Y_{3}^{0}\left(t\right)\right)}^{2}E\left[p_{r}\right]+Y_{3}^{0}\left(t\right){\left(E\left[p_{r}\right]\right)}^{2}-E\left[{\left(Y_{3}^{0}\left(t\right)\right)}^{2}\right]E\left[p_{r}\right]\right)\\ & & -\frac{1}{{\tilde{m}}_{r}}\{-\left(m\chi_{r}+M\alpha_{r}\left(\tau \right)\right)[\omega_{s}^{2}Y_{7}^{0}\left(t\right)+2\zeta_{s}\omega_{s}Y_{8}^{0}\left(t\right)]+[m(\chi_{r}+\frac{\Psi_{L}-1}{L(\tau )}\Pi_{r})+M\Psi_{L}\alpha_{r}\left(\tau \right)]\\ & & \times (-2\zeta_{r}\omega_{r}Y_{4}^{0}\left(t\right)-\omega_{r}^{2}Y_{3}^{0}\left(t\right)+(\omega_{s}^{2}Y_{7}^{0}\left(t\right)+2\zeta_{s}\omega_{s}Y_{8}^{0}\left(t\right))\frac{{\tilde{m}}_{s}}{m_{r}}\int_{0}^{Z_{0}}\Psi \left(\eta \right)dz)\\ & & +2mV\frac{\Psi_{L}-1}{L(\tau )}\chi_{r}Y_{4}^{0}\left(t\right)+\frac{\Psi_{L}-1}{L(\tau )}(mg\chi_{r}+T_{M}\alpha_{r}\left(\tau \right)Y_{3}^{0}\left(t\right)\},\\ c_{3}^{0}\left(Y^{0}\left(t\right)\right) & = & Y_{4}^{0}\left(t\right),\\ c_{4}^{0}\left(Y^{0}\left(t\right)\right) & = & -2\zeta_{r}\omega_{r}Y_{4}^{0}\left(t\right)-\omega_{r}^{2}Y_{3}^{0}\left(t\right)+\left(\omega_{s}^{2}Y_{7}^{0}\left(t\right)+2\zeta_{s}\omega_{s}Y_{8}^{0}\left(t\right)\right)\frac{{\tilde{m}}_{s}}{m_{r}}\int_{0}^{Z_{0}}\Psi \left(\eta \right)dz,\\ c_{5}^{0}\left(Y^{0}\left(t\right)\right) & = & Y_{6}^{0}\left(t\right),\\ c_{6}^{0}\left(Y^{0}\left(t\right)\right) & = & -2\zeta_{M}\omega_{M}Y_{6}^{0}\left(t\right)-\frac{EA}{M}\frac{1}{L(\tau )}Y_{5}^{0}\left(t\right)-\frac{EA}{M}\frac{1}{2L(\tau )}\kappa_{rr}\left(\tau \right)\left({\left(Y_{1}^{0}\left(t\right)\right)}^{2}+2Y_{1}^{0}\left(t\right)E\left[q_{r}\right]-E\left[{\left(Y_{1}^{0}\left(t\right)\right)}^{2}\right]\right)\\ & & -\frac{EA}{M}\frac{\Psi_{L}-1}{L^{2}\left(\tau \right)}\alpha_{r}\left(\tau \right)\left(Y_{1}^{0}\left(t\right)Y_{3}^{0}\left(t\right)+Y_{3}^{0}\left(t\right)E\left[q_{r}\right]+Y_{1}^{0}\left(t\right)E\left[p_{r}\right]-E\left[Y_{1}^{0}\left(t\right)Y_{3}^{0}\left(t\right)\right]\right)\\ & & -\frac{EA}{M}\frac{1}{2}(\frac{\Psi_{L}-1}{L(\tau )}{)}^{2}\left({\left(Y_{3}^{0}\left(t\right)\right)}^{2}+2Y_{3}^{0}\left(t\right)E\left[p_{r}\right]-E\left[{\left(Y_{3}^{0}\left(t\right)\right)}^{2}\right]\right),\\ c_{7}^{0}\left(Y^{0}\left(t\right)\right) & = & Y_{8}^{0}\left(t\right),\\ c_{8}^{0}\left(Y^{0}\left(t\right)\right) & = & -2\zeta_{s}\omega_{s}Y_{8}^{0}\left(t\right)-\omega_{s}^{2}Y_{7}^{0}\left(t\right). \end{array}$$

In further considerations, the original nonlinear system described by Equation (46) is replaced by the linear one expressed by:

(55) $$dY^{0}\left(t\right)=BY^{0}\left(t\right)dt+d\left(t\right)dW\left(t\right).$$

The centralized drift terms are defined as a linear function of the state variables:

(56) $$c_{i,eq}^{0}\left(Y^{0}\left(t\right)\right)=B_{im}Y_{m}^{0},$$

and equivalent coefficients $B_{im}$, determined from the condition of mean-square minimizations the difference between the Equations (51) and (55), are obtained as:

(57) $$B_{im}\kappa_{mj}=E\left[Y_{j}^{0}c_{i}^{0}\left(Y^{0}\right)\right],$$

or in matrix form:

(58) $$B\kappa \left(t\right)=E[c^{0}\left(Y^{0}\left(t\right)\right){Y^{0}}^{T}].$$

The centralized state variables $Y^{0}$ are jointly Gaussian distributed, therefore in further consideration, the relationship for zero-mean Gaussian random vector X, given by [34], is used:

(59) $$E\left[Xf\left(X\right)\right]=E\left[XX^{T}\right]E\left[\nabla f\left(X\right)\right],$$

where non-linear function is denoted by f(X) while ∇ is given by the following expression $\nabla =[\frac{\partial }{\partial X_{1}},\frac{\partial }{\partial X_{2}},\cdots ,\frac{\partial }{\partial X_{n}}{]}^{T}$. Using Equation (57) in transposed form of Equation (56) leads to the formula:

(60) $$\kappa \left(t\right)B^{T}=\kappa \left(t\right)E\left[\nabla {c^{0}}^{T}\left(Y^{0}\left(t\right)\right)\right].$$

Equation (60) describes the components of matrix B as:

(61) $$B^{T}=E\left[\nabla {c^{0}}^{T}\left(Y^{0}\left(t\right)\right)\right].$$

The result of applying Equation (61) to the elements of the centralized drift vector defined by the Equation (54) is matrix *B* obtained as: 

$B=\left[\begin{array}{cccccccc} 0 & 1 & 0 & 0 & 0 & 0 & 0 & 0\\ b_{r}^{\left(1\right)} & -{\tilde{c}}_{r} & b_{r}^{\left(2\right)} & b_{r}^{\left(3\right)} & b_{r}^{\left(4\right)} & 0 & \frac{\omega_{s}^{2}}{{\tilde{m}}_{r}}b_{r}^{\left(5\right)} & \frac{2\zeta_{s}\omega_{s}}{{\tilde{m}}_{r}}b_{r}^{\left(5\right)}\\ 0 & 0 & 0 & 1 & 0 & 0 & 0 & 0\\ 0 & 0 & -\omega_{r}^{2} & -2\zeta_{r}\omega_{r} & 0 & 0 & \omega_{s}^{2}b_{r}^{\left(6\right)} & 2\zeta_{s}\omega_{s}b_{r}^{\left(6\right)}\\ 0 & 0 & 0 & 0 & 0 & 1 & 0 & 0\\ b_{r}^{\left(7\right)} & 0 & b_{r}^{\left(8\right)} & 0 & -\frac{EA}{ML(\tau )} & -2\zeta_{M}\omega_{M} & 0 & 0\\ 0 & 0 & 0 & 0 & 0 & 0 & 0 & 1\\ 0 & 0 & 0 & 0 & 0 & 0 & -\omega_{s}^{2} & -2\zeta_{s}\omega_{s}, \end{array}\right]$ where the particular terms are given by:

$$\begin{array}{ccc} b_{r}^{\left(1\right)} & = & -{\tilde{\omega }}_{r}^{2}-{\tilde{k}}_{r}+\frac{EA}{{\tilde{m}}_{r}}\{\frac{\Gamma_{rr}}{L(\tau )}E\left[u_{M}\right]+\frac{3}{2L(\tau )}\Gamma_{rr}\kappa_{rr}\left(\tau \right)(E\left[{\left(Y_{1}^{0}\left(t\right)\right)}^{2}\right]+E{\left[q_{r}\right]}^{2})\\ & & +\frac{\Psi_{L}-1}{L^{2}\left(\tau \right)}2\alpha_{r}\left(\tau \right)\left(\Gamma_{rr}-\frac{1}{2}\kappa_{rr}\left(\tau \right)\right)(\left(E\left[Y_{1}^{0}\left(t\right)\right)Y_{3}^{0}\left(t\right)\right]+E\left[q_{r}\right]E\left[p_{r}\left(t\right)\right])\\ & & +(\frac{\Psi_{L}-1}{L(\tau )}{)}^{2}(\frac{1}{2}\Gamma_{rr}-\frac{\alpha_{r}^{2}\left(\tau \right)}{L})(\left(E{\left[Y_{3}^{0}\left(t\right)\right)}^{2}\right]+{\left(E\left[p_{r}\right]\right)}^{2})\},\\ b_{r}^{\left(2\right)} & = & \frac{EA}{{\tilde{m}}_{r}}\{\frac{\Psi_{L}-1}{L^{2}\left(\tau \right)}\alpha_{r}\left(\tau \right)\left(\Gamma_{rr}-\frac{1}{2}\kappa_{rr}\left(\tau \right)\right)(\left(E{\left[Y_{1}^{0}\left(t\right)\right)}^{2}\right]+{\left(E\left[q_{r}\right]\right)}^{2})\\ & & +2(\frac{\Psi_{L}-1}{L(\tau )}{)}^{2}(\frac{1}{2}\Gamma_{rr}-\frac{\alpha_{r}^{2}\left(\tau \right)}{L})(E\left[Y_{1}^{0}\left(t\right)Y_{3}^{0}\left(t\right)\right]+E\left[q_{r}\right]E\left[p_{r}\right])\\ & & -\frac{\Psi_{L}-1}{L^{2}\left(\tau \right)}\alpha_{r}\left(\tau \right)\left(E\left[u_{M}\left(t\right)\right]\right)-\frac{3}{2}(\frac{\Psi_{L}-1}{L(\tau )}{)}^{3}\alpha_{r}\left(\tau \right)\left(E\left[{\left(Y_{3}^{0}\left(t\right)\right)}^{2}\right]+{\left(E\left[p_{r}\right]\right)}^{2}\right)\}\\ & & +\frac{1}{{\tilde{m}}_{r}}\{[m(\chi_{r}+\frac{\Psi_{L}-1}{L(\tau )}\Pi_{r})+M\Psi_{L}\alpha_{r}\left(\tau \right)]\omega_{r}^{2}-\frac{\Psi_{L}-1}{L(\tau )}\left(mg\chi_{r}+T_{M}\alpha_{r}\left(\tau \right)\right)\},\\ b_{r}^{\left(3\right)} & = & \frac{2\zeta_{r}\omega_{r}}{{\tilde{m}}_{r}}[m(\chi_{r}+\frac{\Psi_{L}-1}{L(\tau )}\Pi_{r})+M\Psi_{L}\alpha_{r}\left(\tau \right)]-\frac{1}{{\tilde{m}}_{r}}2mV\frac{\Psi_{L}-1}{L(\tau )}\chi_{r},\\ b_{r}^{\left(4\right)} & = & \frac{EA}{{\tilde{m}}_{r}}(\frac{\Gamma_{rr}}{L(\tau )}E\left[q_{r}\right]-\frac{\Psi_{L}-1}{L^{2}\left(\tau \right)}\alpha_{r}\left(\tau \right)E\left[p_{r}\right]),\\ b_{r}^{\left(5\right)} & = & \{m\chi_{r}+M\alpha_{r}\left(\tau \right)-[m(\chi_{r}+\frac{\Psi_{L}-1}{L(\tau )}\Pi_{r})+M\Psi_{L}\alpha_{r}\left(\tau \right)]\frac{{\tilde{m}}_{s}}{m_{r}}\int_{0}^{Z_{0}}\Psi \left(\eta \right)dz\},\\ b_{r}^{\left(6\right)} & = & \frac{{\tilde{m}}_{s}}{m_{r}}\int_{0}^{Z_{0}}\Psi \left(\eta \right)dz,\\ b_{r}^{\left(7\right)} & = & -\frac{EA}{M}(\frac{1}{L(\tau )}\kappa_{rr}\left(\tau \right)E\left[q_{r}\right]+\frac{\Psi_{L}-1}{L^{2}\left(\tau \right)}\alpha_{r}\left(\tau \right)E\left[p_{r}\right]),\\ b_{r}^{\left(8\right)} & = & -\frac{EA}{M}(\frac{\Psi_{L}-1}{L^{2}\left(\tau \right)}\alpha_{r}\left(\tau \right)E\left[q_{r}\right]+(\frac{\Psi_{L}-1}{L(\tau )}{)}^{2}E\left[p_{r}\right]). \end{array}$$

To obtain variances and covariances of particular random state variables the following set of differential equations for covariance matrix $R_{Y^{0}Y^{0}}=E\left[Y^{0}Y^{0}T\right]$ should be solved.

(62) $$\frac{d}{dt}R_{Y^{0}Y^{0}}=BR_{Y^{0}Y^{0}}+R_{Y^{0}Y^{0}}B^{T}+dd^{T},$$

together with the differential equations for mean values defined by the Equation (50). As a result the set of 44 differential equations is obtained that can be solved numerically.

## 3. Numerical Results and Discussion

### 3.1. Main Assumption for Numerical Calculation

In the simulation study and analysis, the case of the lift moving downwards from the building’s top to the base level is considered (see Figure 1a). The main mass of the lift car is assumed to be M=3600 kg and it is attached at the lower end of the cable system comprising $n_{r}=6$ wire ropes (compare Figure 1c). For every rope, the mass per unit length is equal to m=0.872 kg/m, while the longitudinal stiffness is EA=22.889 MN. The values of the transport speed and acceleration taken into consideration are V=2.5 m/s and $a=1\;\;m/s^{2}$, respectively. The total height of the host structure and the initial length of the cable are adopted as $Z_{0}=258.66$ m and $L_{0}=58.66$ m. That brings the value of the travel height H=200 m. The horizontal displacement of main mass is constrained by spring-viscous damping element with the stiffness k=66.689 kN/m and damping coefficient c=9.297 kNs/m. The natural frequency of the main mass is estimated as ${\bar{\omega }}_{M}=\sqrt{k/M}=4.3043$ rad/s, which brings to ${\bar{f}}_{M}=0.685$ Hz, while the fundamental longitudinal natural frequency is defined as $\omega_{M}=\sqrt{EA/ML}$. In this case study, the fundamental mode of the cable-mass system has been used in the single-mode approximation. The damping ratio for the cable lateral mode, the structure damping ratio and damping ratio for the mass lateral and longitudinal mode are assumed as ${\tilde{\zeta }}_{r}=0.003$, $\zeta_{r}=0.025$ and $\zeta_{M}=0.3$, respectively.

### 3.2. Nonlinear Results under Harmonic Excitation via Equivalent Linearization Technique

In nonlinear computation, the ground motion $s_{0}\left(t\right)$ is assumed in the form of harmonic motion defined by the equation $s_{0}\left(t\right)=A_{g}\sin\left(\Omega_{g}t\right)$ where the excitation frequency is adopted as $\Omega_{g}=0.68$ Hz, which is related to the main mass natural frequency. The acceleration magnitude is equal ${\ddot{s}}_{0}$ = 0.1 m/s^2^. Therefore, the amplitude of the ground motion is obtained as $A_{g}={\ddot{s}}_{0}/\Omega_{g}^{2}=0.0055$ m. Using Equation (13) and assuming that $m_{s}\left(z\right)\approx \tilde{m}s=\mathrm{const}$ and $m_{r}=\tilde{m}s\int_{0}^{Z_{0}}\Psi^{2}\left(z\right)dz$ the building response can be described by the formula:

(63) $$p_{r}\left(t\right)=p_{r(max)}\sin\left(\Omega_{g}t-\varepsilon \right),\mathrm{where}\;\;\varepsilon =\tan^{-1}(\frac{2\zeta_{r}r}{1-r^{2}}).$$

For r=1 the phase angle $\varepsilon \to \frac{\pi }{2}$. The amplitude at the top of the building is given by:

(64) $$p_{r(max)}=\frac{A_{g}\int_{0}^{Z_{0}}\Psi \left(z\right)dz}{\int_{0}^{Z_{0}}\Psi^{2}\left(z\right)dz}{\left(R\right)}^{2}\frac{1}{\sqrt{{\left(1-R^{2}\right)}^{2}+4\zeta_{r}^{2}R^{2}}},$$

where $R=\frac{\Omega_{g}}{\omega_{r}}$. In the resonance region, R→1, and then Equation (64) is reduced to the following form:

(65) $$p_{r(max)}=\frac{A_{g}\int_{0}^{Z_{0}}\Psi \left(z\right)dz}{\int_{0}^{Z_{0}}\Psi^{2}\left(z\right)dz}\frac{1}{2\zeta_{r}}.$$

That gives a result of maximum displacement at the top of the structure with a value of 0.15 m. The system of differential equations defined by Equation (31) is then solved numerically by using the 4th–5th order Runge-Kutta algorithm. Time-average of the square of ${\ddot{s}}_{0}$ as the harmonic process is equal.

(66) $$\langle {\ddot{s}}_{0}\left(t\right)\rangle =A_{g}^{2}\Omega_{g}^{4}/2.$$

In the stochastic approach, the ground motion is given by the Equation (44) together with Equation (43). To compare the results obtained from nonlinear solution under harmonic excitation with the expected values obtained by equivalent linearization technique the variance of the stochastic process ${\ddot{s}}_{0}\left(t\right)$, that is given by the following equation:

(67) $$\mathrm{Var}\left({\ddot{s}}_{0}\right)=\omega_{s}^{2}\mathrm{Var}\left(G_{1}\right)+{\left(2\zeta_{s}\omega_{s}\right)}^{2}\mathrm{Var}\left(G_{2}\right),$$

should be compared with the time-average $\langle {\ddot{s}}_{0}\left(t\right)\rangle$. The form of Equation (67) results from the fact that $G_{1}$ and $G_{2}$ are uncorrelated. In the steady-state, the variance is written as follows:

(68) $$\mathrm{Var}\left({\ddot{s}}_{0}\right)=\omega_{s}\frac{\pi P}{2\zeta_{s}}+2\zeta_{s}\omega_{s}\pi P.$$

A comparison of the right hand sides of Equations (66) and (68) leads to the quadratic equation whose discriminant is given by the formula:

(69) $$\Delta =\frac{A_{g}^{4}\Omega_{g}^{8}}{4}-4\omega_{s}^{2}\pi^{2}P^{2}.$$

To satisfy the condition of positive values of quadratic discriminant, the constant spectral density should fulfill the following expression:

(70) $$P\leq \frac{A_{g}^{2}\Omega_{g}^{4}}{4\omega_{s}\pi }.$$

For selected values of *P* the damping coefficients are defined by the formula:

(71) $$\zeta_{s_{1,2}}=\frac{A_{g}^{2}\Omega_{g}^{4}\pm \sqrt{A_{g}^{4}\Omega_{g}^{8}-16\pi^{2}P^{2}\omega_{s}^{2}}}{8\pi P\omega_{s}}.$$

**Figure 3:**
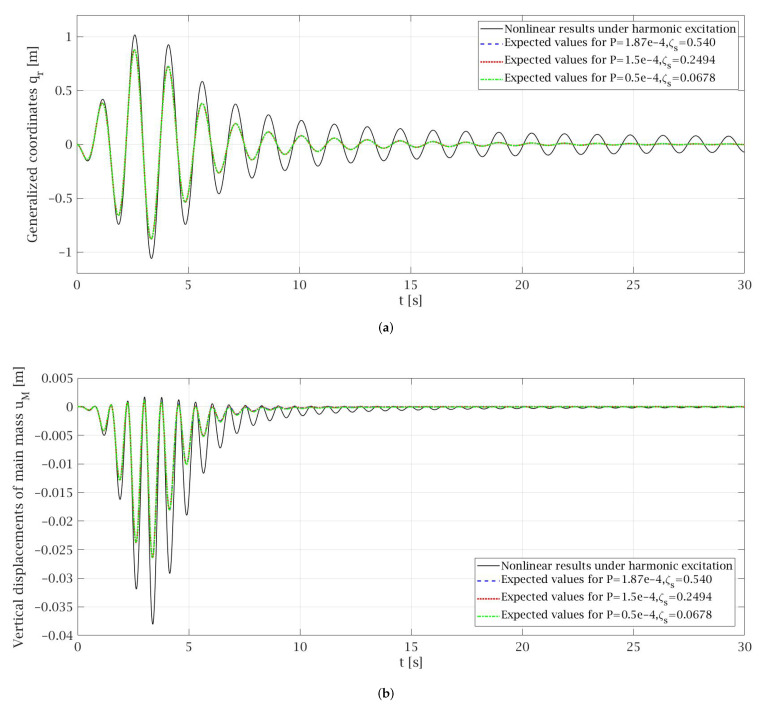
Comparison of the nonlinear results under harmonic excitation with the expected values obtained by equivalent linearization technique: (**a**) Generalized coordinates; (**b**) Vertical displacements of main mass.

**Figure 4:**
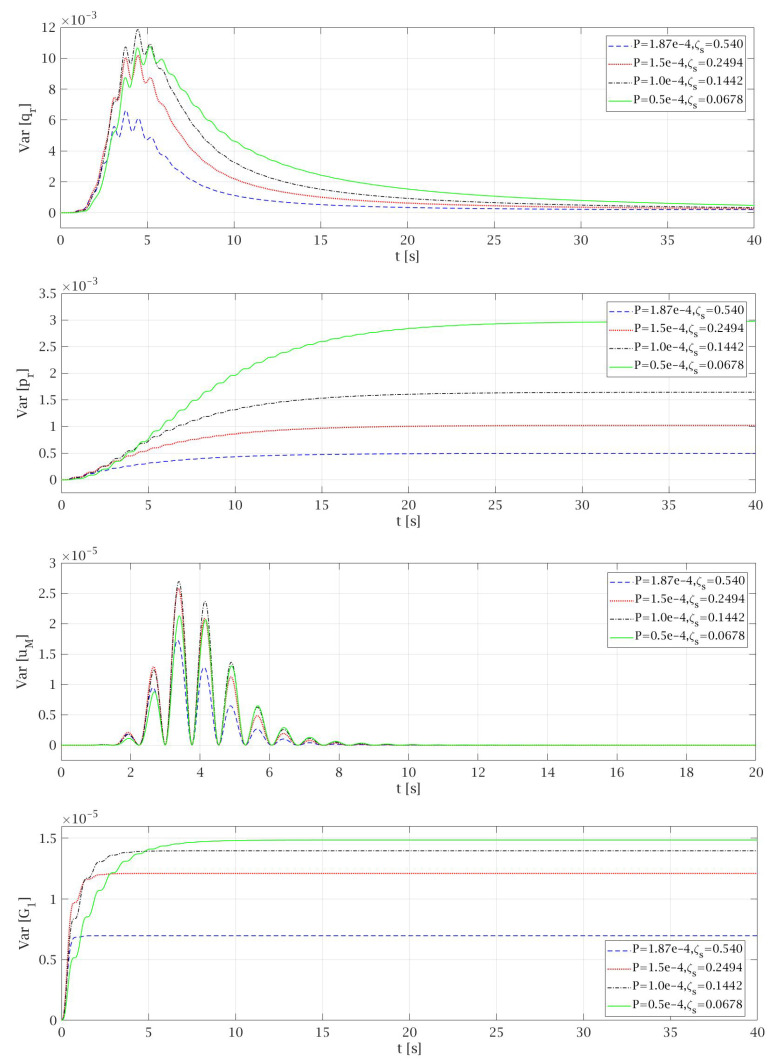
Variances of particular random state variables obtained by the equivalent linearization technique for different values of *P* and $\zeta_{s}$.

Therefore, the highest value of *P* that was taken to the analysis, corresponding to the main data and Equation (70), was 1.87 × 10^−4^ m^2^/s^3^, for which $\zeta_{s_{1}}=0.54$ and $\zeta_{s_{2}}=0.45$ were obtained. The equivalent linearization technique was conducted for different values of *P* and $\zeta_{s}$, that satisfies the Equations (70) and (71), to observe the influence of adopted *P* and $\zeta_{s}$ on the results of expected values (Figure 3) and variances (Figure 4) of particular random state variables. However, for the clarity of presentation only some of them were presented in the paper. Of course, in one process, the value *P* and one of the corresponding damping coefficients $\zeta_{s_{1}}$ or $\zeta_{s_{2}}$ can be considered. The damping coefficient values greater than 1 were omitted in the analysis in advance.

**Figure 5:**
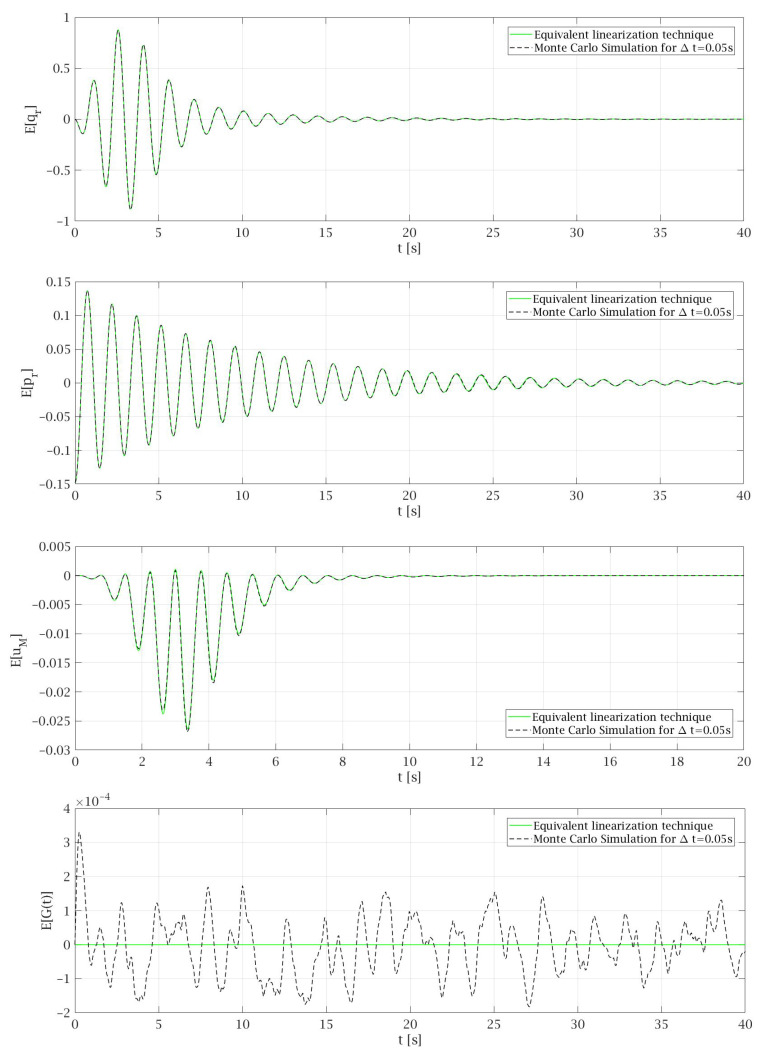
Comparison of the expected values of selected random state variables obtained for *P* = 1.87 × 10^−4^ and $\zeta_{s}=0.54$.

**Figure 6:**
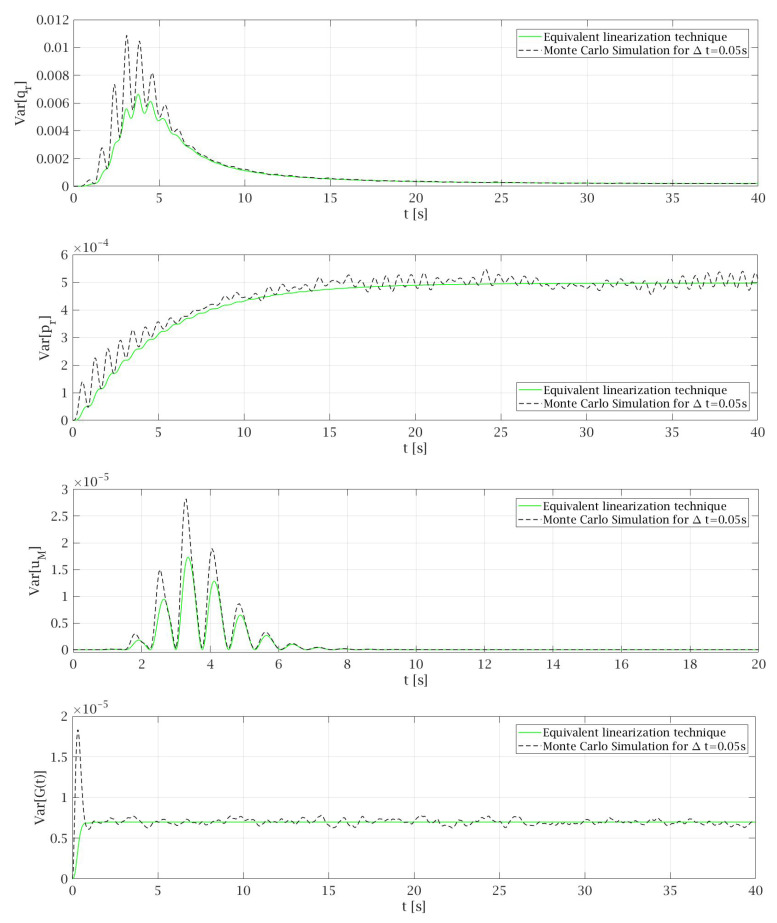
Comparison of the variances of selected random state variables obtained for *P* = 1.87 × 10^−4^ and ζ_s_ = 0.54.

**Figure 7:**
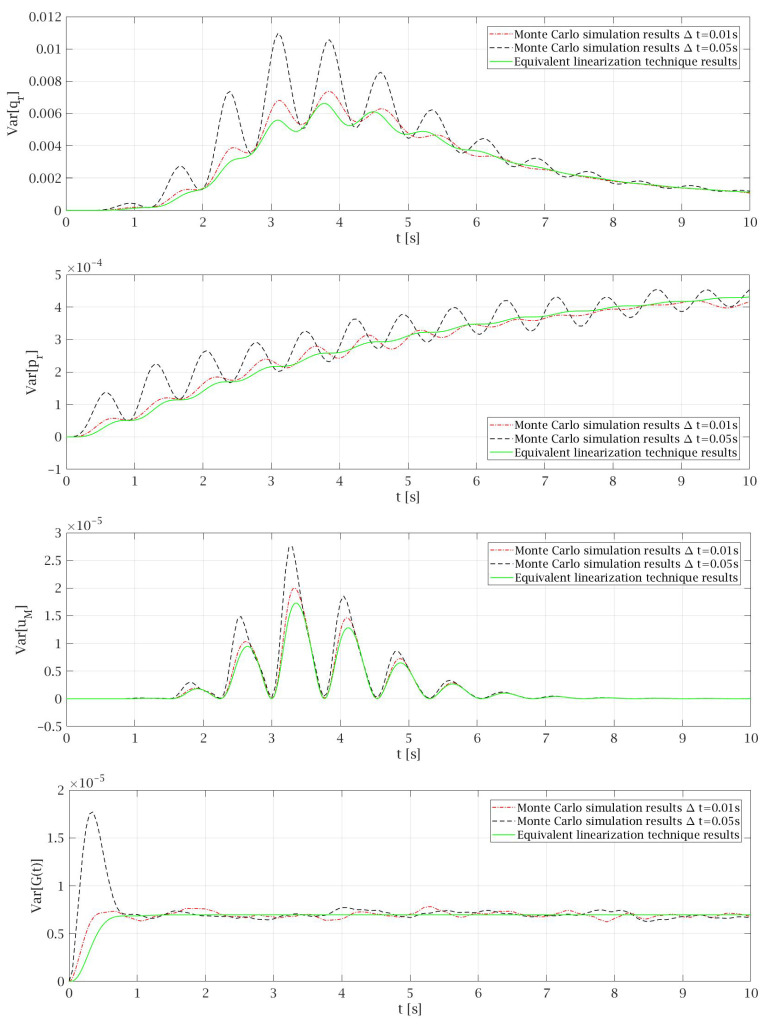
Selected details of the variances of particular random state variables obtained for *P* = 1.87 × 10^−4^ and $\zeta_{s}=0.54$.

The comparison of the results obtained by non-linear solution under harmonic excitation and by an equivalent linearization technique conducted for Gaussian white noise excitation is presented in Figure 3. The total time that is needed for the lift car to travel from the top of the building to the base floor level at the transport speed specified above is about 82 s. However, for the clarity of the presentation, some diagrams presented in Figure 3, Figure 4, Figure 5, Figure 6 and Figure 7 show the selected parts of the motion. In Figure 3 it can be seen that the expected values of the generalized coordinates $E[q_{r}]$ and vertical displacements of the main mass $E[u_{M}]$ are the same for every case of values *P* and $\zeta_{s}$ that were taken into consideration during the analysis. Additionally, the expected values obtained from the equivalent linearization technique are comparable with the deterministic results received under harmonic excitation. The diagrams of the variances for the particular random state variables presented in Figure 4 show that the lower the value of the damping $\zeta_{s}$ is taken into the analysis the higher value of the variance is obtained, which seems to be obvious. Some deviations from this rule can be noticed in the initial phase of the motion.

### 3.3. Verification Using Monte Carlo Simulation

In this section, the results obtained by equivalent linearization technique are compared with the values received from the Monte Carlo Simulation, which was conducted by using 1000 simulations for the different time steps Δt. The set of differential equations that was solved numerically in Monte Carlo simulation by using the 4th–5th Runge-Kutta algorithm is defined by the Equation (45) together with Equations (42) and (36). The most important part in these computation is the correct generation of the white noise process with the zero mean values. Many programs for generating of random variables with the normal distribution and assumed mean value can be found and used in the numerical analysis. The question arises what value of the standard deviation of the white noise excitation process should be assumed in the Monte Carlo simulation in order to be able to compare the results with those obtained by equivalent linearization technique. In many papers information can be found that the Monte Carlo Simulation results depend on the value of the time step Δt taken into the analysis in such a way that, the smaller the step value, the lower the variance. In practice if the value of the standard deviation σ=1 of the white noise excitation process is assumed in the Monte Carlo Simulation the different results of variances of the same variable for different time steps can be observed. It can be seen then that the shape of the lines is similar but the order of magnitude is completely different. So the incorrect assumption of the standard deviation value can lead to misinterpretation of the final results.

As is well known, the differential increment of the Wiener process W(t) may be represented as:

(72) $$dW\left(t\right)=Z\sqrt{dt},$$

where *Z* is the zero-mean Gaussian random variable. Hence, the Gaussian white noise process ξ(t), which is the generalized derivative of the Wiener process, is represented as:

(73) $$\xi \left(t\right)=\frac{dW}{dt}=\frac{1}{\sqrt{dt}}Z.$$

It means that, to make the results of the Monte Carlo simulations independent of the value of the time step Δt, the generated value of the variable *Z* must be divided by $\sqrt{\Delta t}$.

In the Monte Carlo simulation conducted in this work, the standard deviation of the Gaussian white noise process is assumed according to Equation (73) with the value $\sigma =1/\sqrt{\Delta t}$. This leads to the variances that are comparable for every time step Δt. During the analysis, the Monte Carlo simulation was made for different values of *P* and $\zeta_{s}$ and the behavior of the system is similar in each case. Therefore, in this article only results for *P* = 1.87 × 10^−4^ m^2^/s^3^ and $\zeta_{s}=0.54$ that are presented in Figure 5, Figure 6 and Figure 7 are discussed. Figure 5 shows that the expected values of particular random state variables are almost the same in both methods. The only differences can be seen in case of E[G(t)] where in equivalent linearization technique a solid line with the value 0 is observed while in the case of the Monte Carlo simulation, the results oscillate around zero, because they depend on the Gaussian white noise process, which is generated as a sequence of random variables. From Equations (42) and (43) governing the process G(t) it follows that the exact mean value E[G(t)]=0. Since these equations are just appended to the dynamic system and are linear, the equivalent linearization technique yields exact mean value.

In Figure 6, good matches between the variances obtained from the simulations with those obtained by the equivalent linearization technique can be seen. These results were obtained for Δt=0.05 s and only in the transient part of the motion can some differences between both methods be observed. Therefore, Figure 7 shows the variances obtained for the first 10 s of the motion, to depict that if smaller Δt is taken for the Monte Carlo simulation then better matches between the diagrams from both methods can be observed. Additionally, adopting Δt=0.01 s allows us to avoid disturbances in first second of the motion that for the bigger values of time steps lead to incorrect results of Var[G(t)] which are made by inaccuracies due to numerical analysis. On the other hand, the fivefold reduction of Δt brings multiple increase of the time that is needed to conduct the Monte Carlo simulation. What is worth mentioning the equivalent linearization technique is not so sensitive for time step variation because obtained results are very similar for different Δt. Only a significant increase in the time step above quarter of fundamental period of the motion causes disturbances in the obtained values. This remark is important from the point of view of the time that is needed to conduct the computations.

It can be noticed that the values of the variances of particular random state variables depend on the response of the system. As it can be seen in Figure 5 the results of expected values in case of generalized coordinates of cable-mass system $E[q_{r}]$ reach the highest values. On the second position are expected values of generalized coordinates of structure $E[p_{r}]$. Much lower values can be observed in the case of $E[u_{M}]$ and the lowest for E[G(t)]. The same regularity can be observed in the diagrams of the variances of particular random state variables (compare Figure 6). In case of $\mathrm{Var}[q_{r}]$ the obtained results reach the most significant values, for $\mathrm{Var}[p_{r}]$ the results are smaller in comparison to the first one, while the smallest values can be observed in the case of Var[G(t)].

Figure 5, Figure 6 and Figure 7 present good matches between the diagrams obtained by Monte Carlo simulation and equivalent linearization technique, which show that the proposed linearized model is adequate. It leads to the conclusion that the equivalent linearization technique can be successfully used to obtain the mean values and variances of particular random state variables. The most important advantage of this solution is the much lower time needed to obtain results. For example, for small time steps like Δt=0.01 s, the total time to conduct the Monte Carlo simulation is several hundred times longer in comparison to the equivalent linearization technique. The application of the proposed method in the computer codes leads to a procedure that can be easy adapted to objects with different parameters.

## 4. Conclusions

Earthquakes lead to motions of the building base that cause bending deformations of the host structure. This results in vibrations of the top of the building, which cause the excitation of the steel wire cables and ropes inside the lift installation. In the resonance region, the amplitude of the vibration increases significantly. This can cause failure of the lift, excessive fatigue of steel wires and damage of the rope. Lifts are pivotal components of the building infrastructure and to prevent damage/ failure to the installation their behavior needs to be thoroughly investigated. Because the nature of earthquakes is nondeterministic it should be considered by using the stochastic method.

The results presented in this paper show that the proposed model of earthquake excitation represented as a filtered Gaussian stochastic process can be used to replace an idealized deterministic harmonic one. The equivalent linearization technique leads to the replacement of the original non-linear system governed by differential equations with an equivalent linear one governed by differential equations whose coefficients are expressed in terms of expectations of the non-linear functions of the response process. It allows us to obtain mean values, variances and covariances of particular random state variables, which gives a better view of an earthquake’s influence on the behavior of the entire system. This information is very important for the design process. The presented procedure can be easy implemented in computer codes by using numerical techniques. In this approach, the important factor is that the time needed to obtain the results by using the equivalent linearization technique is much lower compared to that for other statistical methods.

## Data Availability

The data presented in this study are available on request from the corresponding author.
